# Morbidité et facteurs de risque de mortalité néonatale dans un hôpital de référence de Douala

**DOI:** 10.11604/pamj.2015.20.258.5648

**Published:** 2015-03-17

**Authors:** Danielle Christiane Kedy Koum, Noel Emmanuel Essomba, Guy Pascal Ngaba, Sintat Sintat, Paul Koki Ndombo, Yves Coppieters

**Affiliations:** 1 Faculté de Médecine et de Sciences Pharmaceutiques, Université de Douala, Douala, Cameroun; 2 Hôpital de District de Bonassama, Douala, Cameroun; 3 Faculté de Médecine et de Sciences Biomédicales, Université de Yaoundé, Yaoundé, Cameroun; 4 Université Libre de Bruxelles, Ecole de Santé Publique, Bruxelles, Belgique

**Keywords:** Mortalité néonatale, Laquintinie, Douala, Cameroun, neonatal mortality, Laquintinie, Douala, Cameroon

## Abstract

**Introduction:**

Cette étude avait pour but d’étudier la mortalité néonatale hospitalière et les facteurs associés, dans un hôpital de référence de la ville de Douala au Cameroun.

**Méthodes:**

Il s'agit d'une étude de cohorte prospective qui s'est déroulée du 1er janvier au 31 avril 2014 dans l'unité de néonatologie de l'hôpital Laquintinie de Douala. Les nouveau-nés de 0 à 28 jours étaient concernés. Les données sociodémographiques, cliniques et l’évolution hospitalière ont été enregistrées. La durée moyenne d'hospitalisation était de 9,9± 6,9. Les tests du Chi2, de Student et une analyse bivariée ont permis de mesurer les associations entre variables. A l'aide de la régression multivariée les facteurs associés à la mortalité ont été déterminés. Le taux de significativité était de 0,05.

**Résultats:**

Au total,350 nouveau-nés ont été inclus, avec un taux de mortalité de 20,3%. L'hyperthermie était le principal motif de consultation avec 102 (29%) patients. Les principales affections associées au décès étaient: les infections 39 (54,9%) (p = 0,0001), la prématurité 31 (43,6%) (p < 0,05), les troubles de l'adaptation 23 (32,4%) (p < 0,005), les encéphalopathies 5 (7%) (p < 0,005) et le paludisme 5 (7%) (p = 0,03). L'on notait comme facteurs associés à cette mortalité, la naissance hors de l'hôpital 51(71,8%) (p< 0,005), la présentation de siège (p = 0,02), l’âge gestationnel < 0,005), l’âge < 1500g (p < 0,005).

**Conclusion:**

Le contrôle des facteurs associés à la mortalité peut conduire à une réduction de la mortalité néonatale.

## Introduction

La mortalité néonatale reste un problème majeur de santé publique dans le monde, et représente plus de 60% des décès des nouveau-nés avant leur premier anniversaire [1]. Près de 6,3 millions d´enfants sont morts avant l´âge de 5 ans en 2013 [2]. La période néonatale précoce est très critique car près des deux tiers de ces décès se déroulent à cette période [3]. La réduction de la mortalité néonatale est impérative pour réduire la mortalité infantile [4]. Au Cameroun, l'Enquête Démographique et de la Santé en 2011 a rapporté un taux de mortalité néonatale global de 31% [5]. Cependant la mortalité néonatale intra hospitalière est plus élevée et varie selon des structures de santé et le niveau de la pyramide sanitaire. Les facteurs influençant les variations de mortalité intra hospitalière sont nombreux: l´environnement socio-économique, l´accès aux soins, le type de patients, le plateau technique et les ressources humaines [6]. Très peu de données publiées sont disponibles en ce qui concerne la mortalité néonatale hospitalière dans la ville de Douala. Kedy Koum et al. ont retrouvé une mortalité néonatale intra-hospitalière de 8% entre 2009 et 2012 à l´hôpital de district de Bonassama, hôpital de catégorie inférieure à l´hôpital Laquintinie de Douala (HLD) [7]. L´hôpital Laquintinie de Douala est un hôpital de référence dans la ville de Douala et aucune donnée relative à la mortalité néonatale intra-hospitalière n´a été publiée à ce jour. Le but de la présente étude était de rapporter la morbidité, le taux de mortalité, ainsi que les facteurs associés à la mortalité néonatale à l´HLD en vue d'améliorer la prise en charge des nouveau-nés.

## Méthodes

**Type, période et cadre d´étude:** il s'agit d'une étude de cohorte prospective dont le recrutement des sujets s'est opéré du premier janvier au 30 avril 2014 dans le service de néonatologie de l´HLD, structure hospitalière de deuxième catégorie située dans la ville de Douala au Cameroun. Le Service comptait: 41 berceaux, 7 couveuses et 7 lits pour la méthode Kangourou. Le personnel était constitué d´un pédiatre, 2 médecins généralistes, 2 infirmières major, et une équipe de 20 infirmiers.

**Population d´étude:** la population d’étude était constituée des nouveau-nés âgés de 0 à 28 jours hospitalisés dans le service de néonatalogie pendant la période d´étude. Un recrutement consécutif a été réalisé. Etaient inclus tous les nouveau-nés hospitalisés dont les parents avaient donné leur consentement. La taille minimale calculée à l'aide de la formule de Lorentz était de 174 sujets.

**Collecte des données:** les données étaient collectées en deux étapes, une interview du parent, suivi de l'examen physique du nouveau-né. Sur la base d'un questionnaire préétabli, testé et restructuré, les parents étaient interviewés. Parmi les variables indépendantes renseignées on distinguait, les données sociodémographiques des mères (âge, situation matrimoniale, niveau scolaire et revenus) et des nouveau-nés (âge, sexe, maternité de provenance), les antécédents maternels (parité, nombre de consultations prénatales, statut vaccinal antitétanique, prophylaxie antianémique et antipaludique, pathologies maternelles); les données liées à l'accouchement (lieu, terme, mode d'accouchement, type de présentation, pathologies maternelles pendant l'accouchement), les données cliniques des nouveau-nés (la température, le poids, le périmètre crânien, la taille, le diagnostic d´hospitalisation, le mode de sortie). L'examen clinique était réalisé à l´admission et tous les jours jusqu´à la sortie du nouveau-né de l'HLD. Le diagnostic était confirmé par un médecin du service. La prise en charge était effectuée selon les protocoles du service.

**Analyse des données:** les données étaient enregistrées et traitées à l'aide des logiciels Excel 2007 et Epi Info 7. et analysées à l'aide du logiciel XL Stat 7.5.2. Les variables quantitatives étaient présentées en moyenne, les variables qualitatives en effectifs et pourcentages. En analyse bi-variée, la comparaison entre les variables qualitatives avait été effectuée à l'aide du test de Chi2. Les variables quantitatives ont été comparées grâce au test de Student. Le test de régression multivariée avait permis de déterminer les facteurs associés à la mortalité. Les différences ont été considérées significatives pour p < 0,05.

**Considérations éthiques:** l’étude a reçu l´approbation du Comité National d'Ethique, les données recueillies ont été analysées de façon anonyme, la confidentialité était de mise.

## Résultats

### Caractéristiques sociodémographiques

Au total, 350 nouveau-nés ont été inclus dans cette étude, 326 (93,1%) en période précoce, dont cent quatre-vingt-quinze (55,7%) le jour même de leur naissance. Le sexe masculin a été majoritaire 195 (56%), avec un sexe ratio de 1,25. Deux cent un (57%) nouveau-nés sont provenus d´une maternité autre que celle de l´HLD. Cent trente-deux (37,7%) mères de nouveau-nés avaient moins de 25 ans, 195 (55,7%) entre 25 et 35 ans. Il a été noté que, 34(10%) mères avaient le niveau d’éducation du primaire, 272 (78%) celui du secondaire, 43 (12%) celui du supérieur. Par ailleurs, 221 (63%) mères étaient célibataires et 221 (63%) mères n´avaient pas de revenus propres.

### Antécédents anténataux et périnataux

La majorité des mères soit 316 (90%) ont effectué les consultations prénatales (CPN) en dehors de l'HLD, 209 (59,7%) ont fait au moins quatre CPN, 196 (56%) ont été primipares et 305 (87%) ont présenté des grossesses monofœtales. Parmi ces femmes, 87 (25%) n'ont pas été protégées contre le tétanos, 346 (99%) ont bénéficié de la prophylaxie anti anémique et 342 (98%) ont reçu le traitement préventif intermittent contre le paludisme (TPI). Le paludisme a été la première pathologie pendant la grossesse. Concernant les antécédents périnataux, le taux de césarienne a été de 24,3% avec pour indication principale la souffrance fœtale 46 (13,1%) cas. Les principaux problèmes notés pendant le travail ont été: la rupture prolongée des membranes 71 cas (20%), le travail prolongé 50 cas (14%), la fièvre sub partu 15 cas (4%). La présentation céphalique a été la plus fréquente avec 311 (89%). Les autres présentations ont représenté respectivement 9,7%, 1% et 0,3% pour les présentations de siège, transverse et de l´épaule. Le liquide amniotique a été clair dans 68% des cas, méconial dans 16% des cas, teinté dans 13% des cas, purée de pois dans 3% des cas.

### Morbidité néonatale

**Figure 1:**
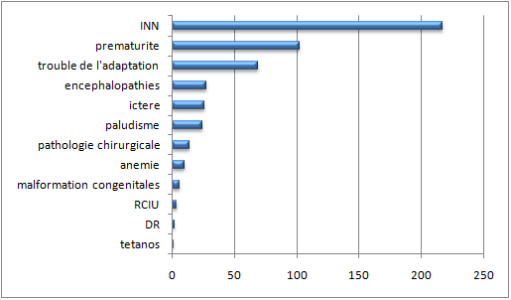
Représentation des pathologies des nouveau-nés hospitalisés

Trente-sept pourcent des nouveau-nés ont été prématurés. L´âge gestationnel moyen a été de 36,6 ± 0,8 semaines, avec des extrêmes de 25 à 44 semaines. Le poids moyen a été de 2622,3g ± 839,1g avec des extrêmes de 800 et 4500g. On notait 36% avec un poids de naissance < 2500g. Parmi eux, 10 (2,8%) ont présenté moins de 1000g. La durée moyenne d'hospitalisation a été de 9,9± 6,9 jours. La température moyenne à l´admission a été de 37,3 ± 0,9°C (35-42°C). L'hypothermie à l´admission a été objectivée chez 50 patients (14%) et l'hyperthermie chez 102 patients (29%). L'infection néonatale a été la pathologie la plus fréquente 217 (62%) (Figure 1).

### Mortalité hospitalière

**Figure 2:**
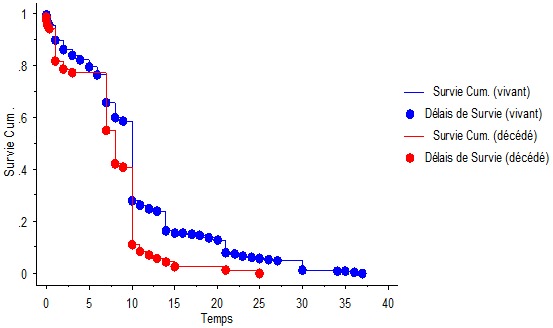
Courbe de survie

Le taux de mortalité a été de 20,3%, avec un taux de mortalité précoce (MNN) de 20,5% et de 16,6% en période néonatale tardive (MNT). La mortalité a été plus élevée chez les nouveau-nés venant des centres autres que la maternité de l'HLD 51/201 (25,3%) par rapport aux nouveau-nés provenant de la maternité de l´HLD, 20/149 (13,4%). Le taux de mortalité a été plus élevé chez les mères célibataires soit 54/221(24,4%) contre 17/129 (13,2%) pour les enfants des mères vivant en couple. En fonction de l’âge gestationnel, 5/6 (83,3%) des nouveau-nés de moins de 28 semaines, 15/53 (28,3%) entre 28 et 31 semaines, 11/71 (15,5%) entre 32 et 36 semaines, 39/215 (18,1%) entre 37 et 41 semaines, 1/5 (20%) à 42 semaines et plus sont décédés. En fonction du poids, 9/10 (90%) nouveau-nés de moins de 1000g, 9/35 (25,7%) entre 1000 et 1499g, 14/80 (17,5%) entre 1500 et 2499g, 39/225 (17,3%) de 2500g et plus sont décédés. La durée moyenne d'hospitalisation était significativement plus élevée chez les vivants que chez les décédés (P= 0,002) (Figure 2).

**Facteurs associés à la mortalité et relatifs aux nouveau-nés:** l´admission en période néonatale précoce, la provenance d´une autre maternité, la présentation de siège, l´âge gestationnel inférieur à 32 semaines, le poids de naissance inférieur à 1500g, influençaient significativement la mortalité du nouveau-né (Tableau 1).

**Tableau 1:** Facteurs liés au décès des nouveau-nés

		Décédé(%)	Vivant (%)	p
Age à l'admission	Précoce	69 (97,1)	245(87,8)	
	Tardif	2(2,8)	34(12,1)	0,03
Sexe	Féminin	28(39,4)	127(45,5)	
	Masculin	43(60,5)	152(54,4)	0,4
provenance	Autre	51(71,8)	150(53,7)	
	Maternité HLD	20(28,1)	129(46,2)	0,009
Mode de présentation	Céphalique	58(81,6)	253(90,6)	
	Siege	13(18,3)	22(7,8)	
	Transversale	0(0,0)	4(1,4)	0,02
Age gestationnel	< 28 SA	5(7,04)	1(0,3)	
	28 - 31 SA	15(21,1)	38(14,07)	
	32 - 36 SA	11(15,4)	60(21,5)	
	37 - 41 SA	39(54,9)	176(63,08)	
	≥ 42 SA	1(1,4)	4(1,43)	<0,0001
Poids	< 1000g	9(12,68)	1(0,35)	
	1000 - 1499g	9(12,68)	26(9,32)	
	1500 - 2499g	14(19,72)	66(23,66)	
	≥ 2500g	39(54,93)	186(66,66)	<0,0001
Température	< 36.5	15(21,13)	35(12,54)	
	36.5 - 37.5	37(52,11)	161(57,71)	
	> 37.5	19(26,76)	83(29,75)	0,1

**Pathologies associées aux décès des nouveau-nés:** les infections néonatales les troubles de l´adaptation et encéphalopathies, la prématurité et le paludisme étaient significativement liés au décès (Tableau 2).

**Tableau 2:** Associations entre les pathologies du nouveau-né et les décès

pathologies des nouveau-nés	Décédé(%)	Vivant(%)	p
**Paludisme**	5(7,0)	19(6,8)	0,030
**Encéphalopathies**	5(7,0)	22(7,8)	< 0,0001
**prématurité**	31(43,6)	99(35,4)	< 0,0001
**Trouble de l'adaptation**	23(32,3)	46(16,5)	< 0,0001
**INN**	39(54,9)	178(64,0)	0,0001

**Facteurs associés à la mortalité et relatifs à la mère:** le statut de mère célibataire et le nombre de consultations prénatales inférieur à 4 influençaient significativement les décès (Tableau 3).

**Tableau 3:** Association entre les facteurs liés à la mère et les décès

		Décède (%)	Vivant(%)	P
**Age**	14 – 24 ans	30 (42,25)	102(36,56)	
	25 - 35 ans	38(53,52)	164(58,78)	
	36 - 42 ans	3(4,23)	13(4,66)	0,6
**Statut matrimonial**	Célibataire	54(76,06)	167(59,86)	
	Mariée	16(22,53)	112(40,14)	0,01
**Niveau d'instruction**	Primaire	6(8,45)	29(10,39)	
	Secondaire	58(81,69)	214(76,70)	
	Supérieur	7(9,86)	36(12,90)	0,6
**Revenu**	Non	44(61,97)	177(12,90)	
	Oui	27(38,03)	102(36,56)	0,9
**parité**	Multipare	14(19,72)	64(22,94)	
	Paucipare	14(19,72)	61(21,86)	
	Primipare	43(60,56)	154(55,20)	0,7
**Lieu de CPN**	HLD	4(5,63)	28(10,04)	
	Autre	67(94,37)	251(89,96)	0,3
**Nombre de CPN**	< 4	40(56,34)	101(36,20)	
	≥ 4	31(43,66)	178(63,80)	0,003
**Mode d'accouchement**	Césarienne	13(18,31)	72(25,81)	
	Voie basse	58(81,69)	207(74,19)	0,2
**Type de grossesse**	Gémellaire	5(7,04)	21(7,53)	
	Monofœtale	66(92,96)	258(92,47)	0,3
**RPM**	Non	60(84,51)	219(78,49)	
	Oui	11(15,49)	60(21,51)	0,3

## Discussion

### Les limites de l’étude

L´étude étant prospective, la majorité des données était bien renseignée. Cependant pour certaines mères transférées des centres de santé environnants, les carnets de consultations prénatales étaient non disponibles, rendant certaines données prénatales et natales inaccessibles.

### Les caractéristiques sociodémographiques

La majorité des enfants (93,1%) sont admis en période néonatale précoce dont 195 (55,7%) à la naissance. Plus de la moitié des nouveau-nés hospitalisés ne poviennent pas de la maternité de l'HLD. L'HLD est l´hôpital de référence ayant la plus grande capacité d'accueil en néonatologie dans la vile de Douala. Sa grande fréquentation est également expliquée par sa situation centrale dans la ville et le coût relativement abordable des consultations pour les populations à faible ressources. Par ailleurs la majorité des mères sont célibataires (63%) et sans revenus (63%). Les enfants des mères célibataires sont particulièrement exposés aux complications périnatales [8].

### Antécédents anténatals

Quatre vingt dix pour cent des mères ont fait leurs consultations prénatales dans une autre structure sanitaire que l´HLD. L´Hôpital Laquintinie est un hôpital de référence pour le suivi et les accouchements des grossesses à risque. On y transfert en principe des femmes ayant fait leurs consultations prénatales dans d'autres formations sanitaires pour des grossesses ou des accouchements à risque, également des nouveau-nés malades. Cent quarante une mères (40,3%) ont fait moins de quatre consultations prénatales. L´OMS recommande de faire au minimum 4 consultations prénatales [9]. Le nombre insuffisant de CPN est significativement lié à une mortalité néonatale plus élevée d´où la nécessité de sensibiliser les mères sur l´importance de ces dernières [10]. Concernant les traitements prophylactiques observés dans la présente étude, la gratuité de la prophylaxie anti palustre expliquerait un taux élevé de prise de médicaments y afférent 342(98%). Cependant malgré ce taux de couverture anti palustre élevé, le paludisme reste en tête des pathologies de la mère en période de gestation. En effet, 34% des mères ont fait le paludisme pendant leur grossesse d´où l´importance d´associer à la prise du traitement préventif, l´usage systématique de la moustiquaire imprégnée à longue durée d´action et des autres mesures de prévention contre le paludisme [11]. Un peu plus d´un quart des femmes n´ont pas correctement été vaccinées contre le tétanos, exposant ainsi leurs enfants au tétanos néonatal. Cette faible couverture vaccinale explique la persistance du tétanos néonatal et l´urgence d´attirer l´attention des femmes enceintes lors des CPN sur l´importance de la vaccination antitétanique.

### Antécédents peri-nataux

En ce qui concerne les pathologies des mères pendant l´accouchement, la rupture prolongée des membranes a été très fréquente 71(20,4%) cas. On notait également des cas de travail prolongé, de fièvres sub partu traitées comme paludisme. Ces différents facteurs augmentent le risque de prématurité, d'infection néonatale, d´asphyxie néonatale et de mortalité néonatale [12, 13]. L´accouchement par césarienne représente 24,3% des cas et sa principale indication est la souffrance fœtale (13,1%). Ce taux de césarienne est plus élevé que celui trouvé par Kedy Koum et al. à l´hôpital de district de Bonassama entre 2009 et 2012 soit 16% [7]. Cependant les césariennes n´ont présenté aucune influence sur la mortalité néonatale, preuve que ces décisions, lorsqu'elles respectent la bonne indication améliorent plutôt la survie du nouveau-né et peut permettre d’éviter 71% des décès périnataux [14].

### Morbidité néonatale

Trente-six pour cent des nouveau-nés ont présenté un faible poids de naissance et 37,1% ont été prématurés. Ces chiffres sont plus élevés que ceux retrouvés dans un travail similaire dans la même ville [7]. Ceci s´explique par le fait que L´HLD soit un centre de référence pour les prématurés et les faibles poids de naissance. L'hypothermie et l´hyperthermie ont été respectivement présentes chez 50 (14%) et 102 (29%) nouveau-nés à l'admission. Ces résultats sont relativement plus bas que ceux de Kedy Koum et al. qui avaient retrouvé respectivement 29,0% de nouveau-nés avec une hypothermie et 35% avec une hyperthermie à l'admission [7]. Des travaux similaires font état de ces trouvailles [1, 15]. Les pathologies du nouveau-né restent pratiquement les mêmes en Afrique Sub-saharienne où seul leur ordre de fréquence varie selon les études [2, 16]. L'infection néonatale reste le motif le plus fréquent d´hospitalisation 62%. Kedy Koum et al. avaient également retrouvé ce profil à l´hôpital de district de Bonassama ou 76,8% des nouveau-nés étaient traités pour une infection néonatale précoce [7]. Les nouveau-nés ont été en général traités sur la base d´une probabilité d´infection néonatale basée plus sur des arguments anamnestiques et cliniques que sur la confirmation bactériologique de l´infection. Les tests inflammatoires tels que la CRP ont été rarement effectués. Les parents doivent payer d´eux même tous les soins de leurs enfants. Les soins néonataux sont assez onéreux et pas accessibles pour la majorité des parents. Les signes de l´infection néonatale étant non spécifiques, il existe donc un risque de sur-traitement avec des implications sur les coûts directs et indirects qui peuvent en découler. De plus il existe un risque accru d´infections nosocomiales, du fait d´une augmentation des séjours hospitaliers injustifiés. Pour diminuer ce risque de sur-traitement, il serait important de mettre en place dans les services de néonatologie des protocoles de prises en charge se basant sur les antécédents maternels, les arguments cliniques, les tests biologiques réalisés au bon moment, avec des critères clairement définis d´arrêt du traitement. Il est également important de faire des mises à jour de l´évolution de l´écologie bactérienne en néonatologie afin d´adapter les traitements de première intention. La prématurité a été un motif très important d´hospitalisation à l´HLD et a concerné 130 (37,1%) nouveau-nés. Mohammad et al. révélaient que le suivi défectueux des grossesses en est l´une des causes fréquente [17]. Dans cette étude 40% des patientes n´ont pas effectué au moins 4 CPN. Ceci renforce le besoin de sensibilisation des femmes communautaires sur l'importance des CPN. En outre, les soins obstétricaux et néonataux essentiels d´urgence (SONEU) et la méthode mère Kangourou validée par l´OMS doivent être vulgarisés à tous les niveaux de la pyramide sanitaire pour réduire la morbidité et la mortalité du nouveau-né et particulièrement celle du nouveau-né prématuré et de faible poids de naissance [7, 18]. Les troubles de l'adaptation 69(19,7%), de l'encéphalopathie 27(7,7%) ont été très fréquents à l´HLD. Ces troubles ont des répercussions immédiates sur la morbidité et la mortalité du nouveau-né mais aussi des conséquences parfois graves sur le développement de l'enfant [19].. Ceci est un appel à la classification des maternités par niveau de compétence en fonction des ressources humaines et de leur plateau technique. C'est également un appel au renforcement des directives visant à transférer les grossesses à risque in utéro vers les centres spécialisés où la mère et le nouveau-nés peuvent être convenablement pris en charge dans une optique de réduction de la mortalité de la mère et de l´enfant [20]. Les SONEU et la réanimation du nouveau-né en salle de naissance doivent être enseignés dans tous les centres qui font des accouchements. L'ictère néonatal a été peu fréquent avec 26(7,4%) cas. Ce nombre est largement sous-estimé car en général l´ictère se déclenche quand les patients sont sortis de l´hôpital. Il est important de sensibiliser les mères sur le dépistage de l´ictère et la nécessité de consulter en cas d´apparition. L´ictère néonatal est souvent physiologique et peu dangereux mais certaines conditions pathologiques peuvent rendre le nouveau-né à risque d´ictère nucléaire avec les conséquences graves sur le développement de l´enfant qui en découlent [21]. Le paludisme a été retrouvé chez 24 nouveau-nés (7%). cette fréquence interpelle par rapport à l´efficacité des mesures prises pour protéger la mère et le nouveau-nés contre le paludisme. Il est donc important d´insister sur l´usage de la moustiquaire imprégnée à longue durée d´action et sur les autres mesures efficaces de lutte contre le paludisme.

### Les facteurs associés à la mortalité néonatale

Le taux de mortalité a été de 20,3%. Ce taux de mortalité est très élevé par rapport à celui trouvé par Kedy Koum 8% [7]. Le taux de MNN à l'HLD est différent de celui trouvé par Chelo et al. dans une maternité de niveau I à Yaoundé 0,5% [21]. Ceci pourrait s'expliquer par les conditions de travail difficile, avec un ratio personnel malade très faible à l'HLD. La mortalité est plus élevée chez les enfants de moins de 7 jours comme dans la plupart des études dans notre milieu [22–24]. Ceci dénote de l´importance des facteurs anténataux et périnataux. La maitrise des SONEU et de la réanimation néonatale devrait réduire cette mortalité. Les nouveau-nés référés des autres structures sanitaires présentent une mortalité significativement plus importante que ceux provenant de la maternité de l´HLD. Ceci pourrait s´expliquer par le fait qu'ils sont pour la plupart référés dans des états cliniques critiques, à l'aide de moyens de transport non appropriés. De plus le manque de moyens financiers des parents pouvant aider à assurer une prise en charge adéquate et immédiate dès l´arrivée, aggrave cette situation. La réduction de la mortalité néonatale doit impliquer plusieurs secteurs. Elle commande nécessairement l´amélioration du statut socio-économique des ménages et l´amélioration de l´accès aux soins des populations y compris l´amélioration des infrastructures routières et des transports [21]. La présentation siège est la plus associée au décès des nouveau-nés (p = 0,02). Ceci vient confirmer les difficultés de management des grossesses, le plus souvent connus face à des présentations de siège dans la pratique courante. Un nombre important de décès est enregistré chez les mères dont l´âge gestationnel était inférieur à 28 semaines d'aménorrhée (p 21]. L'association notée entre le faible poids de naissance et les décès dans cette étude, vient renforcer cette hypothèse. En effet, ces faibles poids de naissance sont le plus souvent rencontrés soient chez des prématurés, soient chez des nouveau-né dont l’évolution de la grossesse avait connu quelques dysfonctionnements. Tout comme le paludisme, on a noté une association entre toutes les autres infections néonatales et les décès, témoignant s'il était encore besoin de la fragilité des nouveau-nés à ces âges, pouvant succomber à la moindre infection [25].

### Facteurs liés à la mère

La corrélation entre le statut de mère célibataire et la mortalité du nouveau-né a été observée (p = 0,01). Ceci venant certainement renforcer l'importance d'un conjoint dans l'organisation des activités de la vie en général. Cette étude a également révélé que les consultations prénatales inférieures à 4 séances, ont été associées à une mortalité néonatale (p= 0,003). Ceci témoigne de l'importance du suivi de la grossesse, dont les paramètres et les interventions sont capital pour une croissance harmonieuse du nouveau-né. Ces résultats suggèrent de renforcer le suivi de la grossesse par l´amélioration des CPN pour une détection à temps des grossesses à risque, et au final une diminution de la mortalité néonatale.

## Conclusion

Les facteurs associés à la mortalité néonatale ont été répertoriés. Il s'agissait entres autres de l'admission dès la première semaine de vie, la provenance d'une autre maternité que celle de l'HLD, l’âge gestationnel inférieur à 28 semaines d'aménorrhée, le poids de naissance inférieur à 1000g, le statut de mère célibataire, et le nombre de consultations prénatales inférieur à 4 séances. Les infections néonatales sont associées aux décès des nouveau-nés Cette étude suggère une augmentation du nombre et de la qualité des CPN pour une diminution de la mortalité néonatale.
